# Neurobiological, Cognitive, and Emotional Mechanisms in Melodic Intonation Therapy

**DOI:** 10.3389/fnhum.2014.00401

**Published:** 2014-06-02

**Authors:** Dawn L. Merrett, Isabelle Peretz, Sarah J. Wilson

**Affiliations:** ^1^Melbourne School of Psychological Sciences, The University of Melbourne, Melbourne, VIC, Australia; ^2^Department of Psychology, Université de Montréal, Montréal, QC, Canada

**Keywords:** Melodic Intonation Therapy, singing, language rehabilitation, aphasia, mechanisms, neuroplasticity, cognitive, mood

## Abstract

Singing has been used in language rehabilitation for decades, yet controversy remains over its effectiveness and mechanisms of action. Melodic Intonation Therapy (MIT) is the most well-known singing-based therapy; however, speculation surrounds when and how it might improve outcomes in aphasia and other language disorders. While positive treatment effects have been variously attributed to different MIT components, including melody, rhythm, hand-tapping, and the choral nature of the singing, there is uncertainty about the components that are truly necessary and beneficial. Moreover, the mechanisms by which the components operate are not well understood. Within the literature to date, proposed mechanisms can be broadly grouped into four categories: (1) neuroplastic reorganization of language function, (2) activation of the mirror neuron system and multimodal integration, (3) utilization of shared or specific features of music and language, and (4) motivation and mood. In this paper, we review available evidence for each mechanism and propose that these mechanisms are not mutually exclusive, but rather represent different levels of explanation, reflecting the neurobiological, cognitive, and emotional effects of MIT. Thus, instead of competing, each of these mechanisms may contribute to language rehabilitation, with a better understanding of their relative roles and interactions allowing the design of protocols that maximize the effectiveness of singing therapy for aphasia.

The relationship between singing and language impairment has been discussed in case studies and in the research literature for hundreds of years. One such case from 1745 CE presented an individual who had a putative stroke in the left hemisphere and was unable to speak, but was able to sing hymns and say certain rhythmic prayers (Dalin, cited in Benton and Joynt, 1960). Reports of many other individuals who were able to sing accurately and fluently with lyrics despite expressive language impairments prompted a study by Yamadori et al. (1977) to investigate singing ability in those with non-fluent (Broca’s) aphasia following stroke or head trauma in frontal regions of the left hemisphere. They found that most of their participants could sing the melody correctly, while about 50% of participants, including some with severe Broca’s aphasia, could sing the lyrics fluently and without errors. This remarkable *dissociation* between singing and language ability was accompanied in the literature by reports of an observed *association* between singing and language recovery. Over the years, clinicians reported the successful use of singing to assist aphasia rehabilitation (for example, Mills, 1904; Backus, 1945; Gerstman, 1964), and this eventually led to the first formalized singing treatment for aphasia – Melodic Intonation Therapy (MIT).

Melodic Intonation Therapy was introduced for English speakers in 1973 by Albert, Sparks, and Helm. Key features of the method include the intoning (singing) of common phrases at a slow pace with left hand-tapping, following a hierarchy of steps that eventually moves from singing to speech (Sparks and Holland, 1976; Helm-Estabrooks and Albert, 2004; Sparks, 2008). MIT has become well-known throughout the world and has been modified extensively by clinicians and researchers, including adaption to many other languages, cultures, and even other disorders of speech and language (for example, Marshall and Holtzapple, 1976; Goldfarb and Bader, 1979; Miller and Toca, 1979; Van Eeckhout et al., 1982; Neumeister et al., 1983; Seki and Sugishita, 1983; van der Lugt-van Wiechen and Visch-Brink, 1989; Popovici and Mihilescu, 1992; Helfrich-Miller, 1994; Carroll, 1996; Carlomagno et al., 1997; Baker, 2000; Bonakdarpour et al., 2003; Hough, 2010; Vines et al., 2011; Conklyn et al., 2012). Yet despite its ubiquity, a number of key questions regarding MIT remain unanswered: How effective is the method? In what contexts does it work? Which components of the method are critical? What mechanisms are involved?

Previous MIT and singing therapy studies have attempted to answer these questions, but have been limited by a number of factors. Both the difficulty in obtaining homogeneous participant samples and the time and resultant cost to implement the MIT protocol have led to a proliferation of case studies or very small patient samples. The heterogeneity of approaches, all of which have been labeled MIT, often prevents direct comparison across these case studies and small samples. Although a significant number of publications now suggest that MIT and some modifications of MIT promote improved language function, the overall quality of this evidence remains poor (Hurkmans et al., 2012; van der Meulen et al., 2012). While the existing research appears to be sufficient to answer the basic question of whether MIT works, the questions of (i) how well it works, in terms of its effect size and in comparison to other treatment options, (ii) when it works, including for which patient groups and treatment protocols, and (iii) why it works, are all still open to debate. Carefully designed studies and randomized controlled trials will provide some of the answers being sought, and several research groups are currently working toward this end (Schlaug et al., 2008; van der Meulen et al., 2012).

In the midst of these unanswered questions, the existing literature provides a significant amount of speculation about which components of the MIT protocol might be essential and what mechanisms of action might be linked to those components. Unfortunately, few studies have attempted to systematically address these issues. Opinions about the utility of the various features of MIT and possible mechanisms of action have been articulated primarily in the discussion sections of relevant research articles. However, as new MIT studies including both behavioral and neuroimaging components have emerged along with relevant findings in both music neuroscience and neurorehabilitation, it would be useful to reassess the existing theories against the available evidence. Several recent reviews have focused primarily on the MIT method (Norton et al., 2009), protocol variations (Zumbansen et al., 2014), and efficacy (Hurkmans et al., 2012; van der Meulen et al., 2012), with somewhat limited discussion of the putative mechanisms of MIT. The aim of the current review is to examine these putative mechanisms in detail, synthesize the existing evidence, and suggest directions for future basic and clinical research.

## Context for This Review

As mentioned previously, the principal components of MIT are melodic intoning (on a minor third or a simple melody), the use of common, formulaic phrases and sentences, left hand-tapping, and slow rhythmic verbalization (usually one syllable per second, although slower durations or more varied rhythms have also been used; see Sparks et al., 1974; Laughlin et al., 1979). Early explanations for the effects of MIT centered around the notion that the musical components of MIT, particularly the intoning, might promote the use of the right hemisphere for language production (Albert et al., 1973) or allow the right hemisphere to better support residual left-hemisphere function (Sparks et al., 1974; Berlin, 1976). However, other possible explanations were put forward, such as the motivational impacts of MIT (Sparks et al., 1974). The originators of MIT were careful to point out that a psychological mechanism could play a role, but was “probably too simplistic an explanation” (Sparks et al., 1974). Their method papers also suggest that notwithstanding some degree of clinical flexibility, adherence to the general methodology, including each of the principal MIT components, is necessary for successful treatment (Sparks and Holland, 1976). Presumably, they felt that each of these components had an important role in the therapy’s effects.

Despite these early views, many discussions of MIT over the past decades have taken a reductionist approach to the therapy, sometimes suggesting that careful research should determine *which* component is responsible for its therapeutic effects. For example, one of the significant debates in the MIT literature is whether rhythm *or* melody is the effective component (or more effective component). The most common finding in both cross-sectional speech facilitation studies and longitudinal treatment studies that attempt to parse melodic and/or rhythmic components is that rhythm, rather than melody, may account for most of MIT’s effects (Boucher et al., 2001; Stahl et al., 2011, 2013). However, although rhythm clearly plays a fundamental role in MIT and the role of melody is still somewhat ambiguous, it may be an oversimplification, or at least premature given the available evidence, to assume that rhythm alone can account for observed treatment effects in their entirety. While the importance of fundamental research to better understand the contribution of individual MIT components should not be underestimated, we believe that a reductionist interpretation of fundamental research should be avoided. For example, given the inherent rhythmicity of singing and the pitch contours intrinsic to rhythmic speech, fully separating the rhythmic and melodic components of MIT may not be possible, thereby limiting the interpretation of studies that compare the effects of melody and rhythm. In addition, potential interaction effects between components, or indirect contributions of components to therapeutic efficacy, may not be accounted for when considering the role of each component separately, especially with a limited number of outcome measures.

In a similar manner, the search for specific mechanisms of action has often been simplified into a contest between two opposing views: right-hemisphere versus left-hemisphere facilitation. For instance, does MIT promote up-regulation of neural activity in the right-hemisphere language homologs or up-regulation of neural activity in perilesional left hemisphere? Since there is some evidence for each of these views, along with several other potential mechanisms, it seems that searching for a single explanatory mechanism that underpins the effects of this therapy is unlikely to be fruitful. It may be that different mechanisms are in operation across different individuals, based on pre-morbid factors (such as genetics and musicianship), lesion factors (such as location, size, time since onset), and syndrome factors (aphasia vs. aphasia with apraxia of speech, dysarthria, etc.). It may also be that various mechanisms are operating synergistically. Within the literature to date, proposed mechanisms can be broadly grouped into four categories: (1) neuroplastic reorganization of language function, (2) activation of the mirror neuron system and multimodal integration, (3) utilization of shared or specific features of music and language, and (4) motivation and mood. We propose that these mechanisms are not mutually exclusive, but rather represent different levels of explanation, reflecting the neurobiological, cognitive, and emotional effects of MIT. The evidence for our proposal and for the various individual mechanisms is reviewed below.

## Neuroplastic Reorganization of Language Function

The use of MIT to facilitate language reorganization in the brain is by far the most discussed putative mechanism. The first attempt to provide a neurobiological explanation for MIT’s effects was the early hypothesis, mentioned above, that the musical components promote right-hemisphere involvement in language processing. This hypothesis was based on behavioral data available at the time that indicated right-hemisphere lateralization for music processing (for example, Kimura, 1964; Bogen and Gordon, 1971). It was supported by the finding that individuals with intact right hemispheres had better outcomes after receiving MIT than those with bilateral lesions (Naeser and Helm-Estabrooks, 1985). Recent functional and structural neuroimaging cases from Schlaug and colleagues also provide some support for this hypothesis. They found an increase in right-hemisphere language activation and improved language production following MIT in two patients (Schlaug et al., 2008). They also reported increased volume of the right arcuate fasciculus, a white-matter tract connecting temporal and frontal language regions, after intensive MIT (Schlaug et al., 2009; Zipse et al., 2012). In addition, MIT combined with anodal transcranial direct current stimulation over the right inferior frontal region (to increase brain excitability) led to greater language improvements than MIT with sham stimulation (Vines et al., 2011). These studies, spanning a number of different modalities, suggest right-hemisphere involvement in MIT-mediated language recovery.

However, a number of other studies have reported contradictory results. A PET study in a group treated with Thérapie Mélodique et Rythmique (TMR), the French version of MIT, suggested that TMR phrases actually led to left-hemisphere language activation, while normal speech led to homologous right-hemisphere activation (Belin et al., 1996). In a magnetoencephalography study of two cases, MIT led to increased left-hemisphere activation in both cases and divergent changes in right-hemisphere activation (Breier et al., 2010). In the individual who showed improvement with MIT, right-hemisphere activation decreased, while in the individual who showed no improvement, right-hemisphere activation increased. This same pattern of divergent functional activation patterns (using pre- and post-fMRI) and language outcomes after MIT was seen in two recent cases reported by Al-Janabi et al. (2014). They found decreased right-hemisphere activation in the individual who showed language improvements, despite the use of excitatory repetitive transcranial magnetic stimulation (rTMS) in the right hemisphere. Furthermore, Laine et al. (1994) described a patient who showed increased left-hemisphere activation after MIT without a right-hemisphere decrease, and this patient did not respond to the treatment. This is consistent with Belin et al.’s (1996) interpretation in their imaging study that right activation reflects maladaptive language processing associated with persistent aphasia.

This debate mirrors a broader ongoing debate in the aphasia literature about the role of the right hemisphere in language recovery. A substantial body of research has shown that areas of the brain that are normally less involved in some language tasks, particularly in the right hemisphere, may be activated to a much greater extent following left-hemisphere insult (for example, Saur et al., 2006; Richter et al., 2008). However, the timing of this right-hemisphere involvement and the extent to which it reflects beneficial functional reorganization are still controversial. Currently, it is thought that right-hemisphere activation occurs commonly in the post-acute phase, with a return to perilesional left-hemisphere activation over the following months reflecting optimal language recovery or successful rehabilitation (Saur et al., 2006). Yet, some imaging studies have shown activation in right-hemisphere language homologs in chronic aphasia. This may be reflective of ongoing disfluency (Naeser et al., 2004), but in some cases, it appears to be predictive of future neuroplastic reorganization and rehabilitation gains (Richter et al., 2008) or even the result of successful rehabilitation (Crinion and Price, 2005).

Such reorganization and its relationship to functional language outcomes appear to be dependent on a number of factors, including the size and location of the lesion and the related severity of aphasia (Marchina et al., 2011; Wang et al., 2013). In the case of a small lesion in the language-dominant (typically left) hemisphere, areas surrounding the lesion may be more likely to take over the function of the affected language region. Alternatively, in the case of a large lesion, homologous regions in the opposite hemisphere may take on language functions (Crosson et al., 2007b). As Schlaug et al. (2009) have argued, using the right hemisphere for language processing might be the only option for individuals who have large left-hemisphere lesions. It seems that both hemispheres can contribute to functional language under some circumstances, whereas activation in either hemisphere can inhibit good recovery in others (Crosson et al., 2007b; Winhuisen et al., 2007; Turkeltaub et al., 2012). Within the right hemisphere of a single individual, some activation could be helpful and other activation detrimental. Evidence suggests that within the inferior frontal gyrus, inhibition of the right pars triangularis using rTMS contributes to language improvement, while inhibition of the right pars opercularis contributes to language disturbance (Naeser et al., 2005; Turkeltaub et al., 2012).

Given the large degree of variability in language reorganization both during spontaneous recovery and following various treatments, the existing contradictory findings in the MIT literature are not so surprising. The cases reported in the literature are far from homogeneous with regard to the time since the lesion, the size of the lesion, or the location of the lesion. In addition, both genetic and environmental factors, such as music training, can influence neuroplastic capacity (discussed in Merrett et al., 2013). If MIT is able to promote neuroplastic reorganization of the language network, it must do so within the context of these individual differences. The same therapy could lead to different patterns of structural and functional neuroplasticity across individuals who had different brain structure and function to start with. A highly relevant example is the way that the relationship between the singing and language networks in the brain is modulated by singing expertise (Wilson et al., 2011). Since MIT is a singing-based therapy, this variable relationship between the singing and language networks could potentially influence both the efficacy of MIT and the resulting language reorganization. Unfortunately, singing expertise has not typically been thoroughly evaluated in MIT studies to date.

It should also be noted that the results of neuroimaging studies of aphasic language function, both within and outside the MIT literature, should be interpreted in light of the type of language task used for functional imaging and the therapy protocol. A significant body of evidence (reviewed in Van Lancker Sidtis, 2012) indicates that formulaic language production depends on right-hemisphere and subcortical regions, in contrast to the generation of more spontaneous language, which typically depends on the left hemisphere. Formulaic language includes common, highly stereotyped expressions, which are generally used contextually and stored as a unit in memory (Van Lancker Sidtis, 2012). Differences in the degree of formulaicity in functional imaging tasks both between and within studies may significantly impact the lateralization of activation. The use of non-propositional language tasks during functional imaging, such as counting or repeating everyday phrases, may lead to greater right-hemisphere activation than tasks that are more generative in nature. Stahl et al. (2013) suggested that these task-based differences in language lateralization may account for the existing imaging findings. More generally, they also proposed that the use of right-hemisphere language regions could be a function of intensive training of formulaic phrases in MIT, providing an alternative hypothesis to that of music-based promotion of right-hemisphere activation. Formulaic phrases, such as “good morning,” “cup of coffee,” and “How are you?” are often used in the early stages of MIT, and these may be the only phrases that are trained in individuals with severe aphasia who are unable to progress to more complex material. Even if the MIT phrases in a given protocol include less formulaic material, such phrases may become like speech formulas over time with intense repetition. Although the MIT protocols discussed by Sparks (2008) and Helm-Estabrooks and Albert (2004) suggest using a broad range of material to ensure that there is little repetition, the phrases used in MIT are typically highly repetitive in practice. In conjunction with the individual differences mentioned above, the role of formulaicity may explain many of the disparities in previous neuroimaging studies.

It has often been assumed that MIT must have a common mechanism (across all treated individuals with aphasia) by which it promotes language reorganization, such as the exploitation of right-hemisphere music processing regions for language or the use of right corticostriatal formulaic language circuits. While it is likely correct that MIT is effective in activating any intact brain regions that are involved in music processing (both right *and* left) as well as those involved in formulaic language, the assumption that there is a common neuroplastic mechanism and/or that this mechanism is musical or linguistic in nature may be flawed. Rather than depending on the musical or linguistic components to promote a specific type of language reorganization, it may be that MIT can help to promote neuroplasticity of the language network more generically, simply because it allows individuals with aphasia to practice language production intensely. Evidence suggests that treatments that promote intense, complex practice can effectively induce neuroplasticity (Green and Bavelier, 2008; Kleim and Jones, 2008). Other aphasia rehabilitation strategies that have demonstrated some positive effects, such as intensive language–action therapy, are based on such principles (Difrancesco et al., 2012). Furthermore, a significant relationship between intensity and speech and language outcomes was found when existing treatment studies were reviewed (Bhogal et al., 2003). MIT may make language production easier (discussed further below) and thereby encourage intense practice, which could in turn lead to training-induced reorganization.

In sum, evidence from a variety of neuroimaging studies demonstrates that MIT can promote both functional and structural neuroplasticity. It remains unclear how induced neuroplastic change interacts with individual patient characteristics and whether this neuroplasticity is directly related to specific components of the therapy. It is worth noting that the recommended “ideal candidate” for MIT has a language profile that includes poor repetition, paucity of output, and stereotypic utterances (Sparks et al., 1974). Given this profile, the ideal candidate for MIT is likely to be an individual with severe aphasia and a large anterior left-hemisphere lesion. However, many MIT studies are carried out with participants who do not meet the criteria for ideal candidates and who have large variations in lesion size and location, including those with small lesions and only mild to moderate non-fluent aphasia. Different mechanisms may be involved across individuals who have excellent responses to MIT and/or meet the ideal candidate profile versus those who only show a partial response or have different language impairment profiles. The relationship between neuroplastic mechanisms, individual factors, and clinical outcomes needs further exploration. In addition to advancing our understanding of brain plasticity and individual differences, future work addressing these questions will be of great value clinically.

## Observation, Imitation, Integration, and the Mirror Neuron System

Melodic Intonation Therapy is a multimodal therapy, as the therapist provides both an auditory and visual model for the patient, and the protocol contains elements of observation, imitation, and synchronization. A number of different hypotheses have been raised as to how these aspects of the therapy might explain its effects, although these have not been subjected to direct empirical investigation. These hypotheses include: (1) a proposal by Schlaug et al. (2008) that the left hand-tapping used in MIT engages a right sensorimotor integration network in which hand and articulatory movements are closely linked and (2) a proposal by Racette et al. (2006) that the synchronized singing in MIT could promote activation of an “auditory–vocal interface” to improve articulatory motor function. What links these hypotheses together as a category of putative mechanisms is their connection to integration/association functions of the brain and possibly the human mirror neuron system.

Left hand-tapping has been considered a crucial component of the MIT protocol since its inception, although a number of cases have successfully used a modification of MIT without the tapping (for example, Hough, 2010). In their case study, Goldfarb and Bader (1979) demonstrated improvements in phrase repetition using intonation alone compared to normal speech, but hand-tapping appeared to further improve performance. A number of potential mechanisms have been proposed for this MIT component, including enhancement or reinforcement of the rhythmic aspects of MIT and pacing of speech (both discussed below), as well as the up-regulation of right-hemisphere activity related to articulation through sensorimotor coupling. From theoretical, neurophysiological, and behavioral perspectives, speech and language are strongly linked to hand motor control (Meister et al., 2003, 2006; Binkofski and Buccino, 2004; Gentilucci and Dalla Volta, 2008). Based on such findings, Schlaug et al. (2008) have hypothesized that left hand-tapping could activate a right-hemisphere sensorimotor network that is used for articulatory movement. Articulation is often impaired in individuals with non-fluent aphasia because of comorbid motor speech disorders such as apraxia of speech and dysarthria. Given the close proximity of oral and hand movement representations in the motor control system, Schlaug et al. proposed that hand-tapping could lead to a priming effect for orofacial and articulatory movements. Lending indirect support to the idea, an unrelated study has demonstrated that completing a complex, non-symbolic left hand movement in conjunction with naming led to improved performance and increased right-hemisphere activity in aphasic individuals (Crosson et al., 2007a, 2009). The reasoning behind this treatment was that it might activate intention mechanisms in the right frontal lobe and thereby prime right-hemisphere language activity. Another proposal regarding hand-tapping is that the sound of the tapping may promote sensorimotor integration, i.e., a neurobiological coupling between the sound and the co-occurring hand and articulatory actions (Lahav et al., 2007; Schlaug et al., 2008). Such sensorimotor integration has often been linked theoretically and neuroanatomically to the putative mirror neuron system (Lahav et al., 2007).

Mirror neurons are neurons that exhibit multimodal response properties – they are stimulated by certain actions whether those actions are being performed or being perceived (visually or aurally). Recent work, such as Mukamel et al. (2010), demonstrates that neurons with mirror properties occur widely throughout the brain; however, it is widely held that humans have a “mirror neuron system” which consists of specific neural regions including the premotor cortex, inferior frontal gyrus, and inferior parietal areas (Iacoboni and Mazziotta, 2007). While the functions (and even the existence) of a mirror neuron system in humans have been hotly debated, the evidence appears strong that inferior frontal and inferior parietal regions, among others, are activated both in the observation (seeing and/or hearing) and the execution of known actions (Buccino et al., 2001; Gazzola and Keysers, 2009). Such findings have been enthusiastically applied in clinical neuroscience rehabilitation paradigms (Ertelt et al., 2007; Celnik et al., 2008; Bang et al., 2013). For example, Ertelt et al. (2007) combined physical practice with action observation of purposeful hand and arm movements (using video) for upper arm rehabilitation after stroke. They found a significant improvement over controls who completed physical practice only. The results have been attributed to activation of the mirror neuron system, particularly after neuroimaging of object manipulation before and after action observation treatment showed increased activity in parieto-frontal areas considered core regions of the system.

Whether there is an actual mirror neuron system or a more general perception–action integration network in the brain, this mechanism has been proposed to explain the positive effects of MIT (Racette et al., 2006; Overy and Molnar-Szakacs, 2009). The MIT protocol provides the patient with a visual and auditory model to observe, to imitate, and to synchronize with. If observation, imitation, and synchronization of singing or intoned speech are interacting with a neural perception–action integration system, they might be expected to impact motor aspects of speech most strongly (Fadiga et al., 2002; Wilson et al., 2004). Indeed, some of the benefits of MIT are perhaps attributable to improvements in speech articulation (Sparks and Holland, 1976; Wilson et al., 2006) that subsequently lead to improvements in language output. Racette et al. (2006) compared word production and intelligibility in individuals with aphasia when singing and speaking both alone and with an auditory model. They found that choral singing (with a model) led to better word intelligibility than singing alone or choral speaking. Although the advantage of choral singing over choral speaking may be explained at least in part by the slower rate of production in singing than in natural speech, there is still a distinct advantage for singing along compared to singing alone that is unrelated to tempo. The authors suggest that this may be due to activation of a right-hemisphere “auditory–vocal interface” or mirror neuron system, as the improvements appear to depend on the opportunity to sing together and synchronize with an auditory model.

Such a mechanism would not be specific to MIT or singing, but rather, would apply more generally to any speech/language therapy that provides similar multimodal modeling or synchronization opportunities. Fridriksson et al. (2012) recently found that mimicking an auditory–visual speech model induced significantly greater speech output and fluency than an auditory-only model or spontaneous speech in a group of individuals with non-fluent aphasia and concomitant apraxia of speech. If this mechanism alone could account for MIT’s effects, MIT may not offer benefit beyond other multimodal therapies. However, Racette et al. (2006) suggested that the left-hemisphere lesions that typically lead to aphasia may impair the left-hemisphere auditory–vocal interface involved in generative speech, while the intact right-hemisphere auditory–vocal interface may be more responsive to singing or formulaic speech. If so, this could explain why MIT, which includes singing common phrases, would be better placed than other therapies to take advantage of such a system. It is worthwhile noting that singing or intoning activates a bilateral fronto-temporal network that overlaps with the putative mirror neuron system to a certain degree (Ozdemir et al., 2006; Kleber et al., 2007; Wilson et al., 2011). Nonetheless, there is no direct evidence that MIT leverages this system through intonation or hand-tapping. Further investigation into the role of the mirror neuron system in singing, in articulatory motor function, and in language rehabilitation more generally is clearly warranted and may provide insight into the neurobiological mechanisms underlying MIT.

## Shared or Specific Features of Music and Language

One of the current debates in the literature is the extent to which music and language overlap in terms of their neural representation and processing. While differences between the two cannot be denied, there are features that are shared at least superficially by music and language, such as pitch, rhythm, timbre, and syntax (reviewed in Patel, 2008). These shared features have prompted proposals that there could be common processing pathways for music and language, such as Patel’s shared syntactic integration resource hypothesis (Patel, 2003). The idea of common processing pathways for language and music provides a potential cognitive mechanism for MIT that is clearly linked to some of the neuroplasticity hypotheses discussed above. MIT could take advantage of the shared features of music and language, such as pitch and/or rhythm, to access language indirectly through music processing pathways. This is a somewhat controversial proposal. For example, there is significant neuropsychological evidence for modularity of the two systems, with evidence of clear dissociations between language impairment and music impairment (Peretz and Coltheart, 2003; Peretz, 2009). Logically, the more cognitive overlap between music and language, the more likely that dysfunction in the language system would be accompanied by dysfunction in music processing as well. To date, a fully coherent explanation is lacking for how intoning or singing could overlap cognitively with the language network in such a way that it would be independent enough to remain intact despite damage to the language network but interdependent enough to take on language function.

Two possible arguments for this mechanism come from the research literature comparing speaking and singing. First, both speaking and singing are known to be processed bilaterally in the brain, using proximal regions that appear to overlap to a large degree, but with speaking more left lateralized and singing more right lateralized (Jeffries et al., 2003; Brown et al., 2006; Callan et al., 2006; Ozdemir et al., 2006). It appears that sung word production may be less reliant on the left-hemisphere language network than spoken words, even when lyric type and tempo are taken into account. This difference in lateralization may provide the means whereby language functions could co-opt relevant right-hemisphere regions of the singing network in the presence of a left-hemisphere lesion. However, this is difficult to reconcile with the bulk of the neuroimaging findings after MIT treatment presented above. Another study that has investigated the neurocognitive relationship between singing and speaking provides an alternative argument by considering the role of expertise (Wilson et al., 2011). These researchers found that singing expertise is associated with a decoupling of the singing network from the language network, with more focal, left lateralized functional activation for singing that is proximal but posterior to language activation. When considered in conjunction with putative neuroplasticity mechanisms, this raises a number of hypotheses, including (1) that MIT would be more effective in individuals with previous singing experience who have already developed a specialized singing network or (2) that through regular singing practice, MIT could promote the development of a more “expert” singing network that would occupy left-hemisphere perilesional regions. The first hypothesis is indirectly supported in the existing literature, given that Wilson et al. (2006) found that MIT was more effective than rhythmic speech in their case study of a trained musician, while Stahl et al. (2013) did not find an advantage of singing over rhythmic speech in a group of non-musicians. Additional studies are needed to disentangle the relationship between music and language in aphasia and in MIT relative to expertise. Despite being poorly understood, it is possible that an intact singing network would best facilitate language production.

Another set of hypothesized mechanisms steers clear of this debate about shared cognitive processing and simply suggests that specific features of music and/or language can facilitate speech production. A range of possible beneficial effects of the melodic and rhythmic components of MIT has been suggested. For example, Racette et al. (2006) suggested that singing or intoning phrases may provide more time for motor planning and execution than normal spoken language. This could make production more fluent and allow less demanding rehearsal. Lending support to this idea, Laughlin et al. (1979) showed that longer syllable lengths in MIT increased the number of correct phrases produced by patients with non-fluent aphasia. Other studies in dysarthric speakers have indicated that pacing and intervention techniques that reduce speech rate can improve intelligibility, although the exact relationship between speech rate and intelligibility is uncertain (for example, Yorkston et al., 1990; Pilon et al., 1998; Hustad et al., 2003). It may be that the slower articulation of singing benefits some patients, while being less helpful for others (Racette et al., 2006). In another example of a possible effect of melody, Wilson et al. (2006) found a long-term benefit for the production of rehearsed phrases that had a melodic and rhythmic component over those with only a rhythmic component in a musically-trained individual with aphasia. They proposed that the melodic component may have promoted separate representation in memory, leading to superior phrase encoding and retrieval.

Other rhythmic aspects of MIT have also been implicated as facilitators. In the TMR protocol (French version of MIT), word accentuation is greatly emphasized, despite the fact that French does not have the language element of lexical stress, creating a strong sense of rhythm (Van Eeckhout et al., 1982). Singing may be more rhythmic than speech, at least in French. The hand-tapping and steady rhythm used in MIT could also act as a metronome, as pacing is known to be beneficial with articulatory impairments (Brendel and Ziegler, 2008). In their study of the facilitatory effects of singing on aphasic speech, Racette et al. (2006) suggest that increased temporal regularity may be an alternative or additional explanation as to why singing along with a model is more beneficial than speaking along in a syllable-timed language such as French. As a final point regarding rhythmic facilitation, Stahl et al. (2011) suggested that rhythm may be particularly useful in facilitating speech for aphasic individuals who have large basal ganglia lesions. The benefits of rhythm for speech production were evident in this group, whereas a group with no or small lesions in the basal ganglia did not show a rhythmic facilitation effect, suggesting once again a possible interaction between mechanisms and patient variables such as lesion size and location.

In addition to musical features such as melody and rhythm that might act as facilitators, the use of a specific type of language within the therapy may also play a significant role. In the early stages of MIT, most therapists use common, high-probability phrases (Helm-Estabrooks and Albert, 2004). Although the stated goal of the therapy is to improve generative language, the incorporation of formulaic phrases into a functional vocabulary for the patient may become a treatment objective in and of itself, particularly for individuals with severe aphasia. This has been described as palliative use of MIT by Zumbansen et al. (2014). Whether or not the restoration of generative language function is the goal, the use of formulaic phrases may facilitate language by tapping into corticostriatal regions implicated in formulaic, non-generative language (Van Lancker Sidtis, 2012). This language feature may also interact with a number of putative mechanisms of action, including promoting the use of right-hemisphere language regions (as discussed above, Stahl et al., 2013) and motivating patients (discussed below).

## Motivation, Mood, and Arousal

Although regarded as “probably too simplistic an explanation” (Sparks et al., 1974), a potential role for psychological or emotional mechanisms in the efficacy of MIT should not be discounted. These putative mechanisms have received far less attention in the MIT literature, but indirect evidence suggests that they may be highly significant. Singing is a pleasurable and non-threatening way for individuals with aphasia to express themselves vocally, which may help to enhance motivation to continue with an intensive therapy regimen (Racette et al., 2006). A substantial literature exists regarding the use of music as a motivator in sport and exercise, where it can lead to increased output and endurance (Karageorghis and Priest, 2011). This may also occur in the rehabilitation domain, as internal motivation has been shown to be a strong predictor of rehabilitation adherence (Chan et al., 2009). Music therapy has even been used successfully with mental health clients with low motivation for other therapies (Gold et al., 2013). Such studies imply that music might be intrinsically motivating. Neurobiological evidence for a relationship between music and motivation comes from studies showing that pleasurable experiences during music listening activate the brain’s reward/motivation circuitry (Blood and Zatorre, 2001; Menon and Levitin, 2005) and are associated with striatal dopamine release, a neurotransmitter associated with pleasure, motivation, and reward (Salimpoor et al., 2011). Outside of the music domain, the use of formulaic phrases in the early stages of MIT might also enhance motivation, given that these are usually highly familiar and desirable phrases to rehearse, and may even be chosen in conjunction with the patient. Although motivation has not been studied directly in MIT, our own experience is that patients with aphasia report being highly motivated by MIT and have been able to successfully complete intense daily therapy sessions.

As a musical form of language rehabilitation, MIT could potentially harness not only music’s capacity to engage and motivate, but also its ability to influence mood in a positive direction (Pelletier, 2004; Västfjäll et al., 2012). Simply listening to music has been shown to improve negative mood in both healthy adults (Boothby and Robbins, 2011) and in stroke patients (Särkämö et al., 2008; Kim et al., 2011). Active music making, such as singing, also increases positive mood, decreases negative mood, and positively influences biochemistry (Kuhn, 2002; Unwin et al., 2002; Grape et al., 2003; Kreutz et al., 2004). Although it has not been empirically assessed to date, the influence of MIT on mood and motivation may explain some of its efficacy. The use of rehabilitation therapies, such as singing, that can jointly influence both language function and mood might be of great import in the treatment of post-stroke aphasia, since low mood and clinical depression are common comorbidities of stroke (Robinson, 2003; Berthier, 2005).

## Conclusion

The various mechanisms discussed above provide possible explanations of MIT’s effects, spanning neurobiological, cognitive, and emotional domains. Previous discussions regarding MIT have often presented these mechanisms as competing hypotheses, requiring a definitive answer as to which (one) mechanism is causal. However, given the direct evidence for many of these hypotheses and the indirect evidence for others, we take the opinion that, broadly speaking, these are different levels of explanation rather than competing explanations, and they reflect the diverse ways that MIT and its various components can influence speech and language rehabilitation. In almost every case, these are not mutually exclusive hypotheses, and each could contribute to the overall effect of MIT.

This may explain why MIT has been considered an effective treatment option by many clinicians, despite the lack of carefully controlled evidence and the uncertainty as to the mechanisms involved. As mentioned previously, other speech and language therapies have been developed that are based on or explained by many of the mechanisms discussed here, including constraint-induced aphasia therapy, a form of intensive language–action therapy (Pulvermüller et al., 2001; Difrancesco et al., 2012), speech entrainment (Fridriksson et al., 2012), and intention treatment (Crosson et al., 2009). The reported success of these treatments lends credibility to the proposal that similar mechanisms underlie successful treatment with MIT. However, unlike therapies with a single target mechanism, MIT may be uniquely placed to take advantage of many of these mechanisms of action simultaneously. There are three potential implications of this that will be discussed here and that we believe should be the focus of future research.

First, the use of multiple mechanisms could have an additive effect, making MIT a more efficient and/or effective treatment than therapies that target one mechanism. Ideally, the overall effectiveness of MIT compared to other treatment options would be evaluated with large-scale randomized controlled trials, some of which are reportedly underway. Yet, given the difficulty in obtaining this kind of evidence in heterogeneous aphasia populations, other methodologically rigorous methods of comparing MIT efficacy to that of other therapies should be sought. Using research participants with aphasia as their own controls is one possible option. The major caveat to this approach is the potential for carry-over or delayed treatment effects, but careful designs should minimize the problem. Despite concerns regarding generalizability to the larger clinical population, even single cases can help to address this issue if the study designs and statistics used are appropriate (Howard, 1986; Beeson and Robey, 2006). Few studies to date have directly compared MIT with other treatments, and statistical analysis and effect sizes have typically not been included in MIT case studies or case series. These shortcomings in the existing literature should be rectified in future studies so that questions about whether MIT is a more effective treatment can be appropriately addressed.

Second, the use of a variety of mechanisms could make MIT a more flexible treatment for a larger variety of patients, with the use of different mechanisms dependent on individual patient variables. As noted above, MIT was initially designed to treat non-fluent aphasia patients with a specific language profile; however, MIT has now been used to treat a large number of different speech and language disorders, particularly apraxia of speech and disorders of articulation. Furthermore, MIT has shown benefits to patients with vastly differing lesion locations, lesion sizes, severities of aphasia, and language profiles (but see Zumbansen et al., 2014, for a dissenting view). It may be that the wide variety of mechanisms of action confers flexibility on the therapy, making it functional for a number of different disorders or language profiles that would benefit from different mechanisms. A “one-size fits all” approach to speech and language therapy is unlikely to be fruitful and thus is not particularly desirable, whereas clinical constraints and practical considerations would suggest that broadly applicable therapeutic techniques are of value.

Third, the various proposed mechanisms of action in MIT could have a synergistic effect. Evidence from the basic neuroscience literature suggests likely interactions between the various mechanisms implicated in MIT. For example, neuroplasticity is negatively influenced by stress and depression (reviewed in Pittenger and Duman, 2007). As mentioned previously, mood disorders are often comorbid with post-stroke aphasia. If MIT is able to positively influence mood, then treatment-induced neuroplasticity may also be enhanced. Koelsch (2009) has also suggested that emotional processes modulate mirror neuron system activity, potentially linking these two putative MIT mechanisms. Other examples, already discussed elsewhere in this review, include the relationship between cognitive and neurobiological mechanisms and the role of motivation in facilitating intense training that could mediate neuroplasticity. Both the specific musical features of MIT and the communicative content, such as formulaic phrases, may interact with motivation and mood mechanisms. In short, these neurobiological, cognitive, and emotional mechanisms could certainly influence each other, and may lead to different, and perhaps greater, treatment effects than if they were to act in isolation.

Consideration of the mechanisms involved in MIT leads to many questions that can and should be further investigated, including the nature of MIT-induced neuroplasticity, the role of the mirror neuron system, the interaction between underlying cognitive processes for music and language, the role of phrase formulaicity, the relative contribution of mood and motivation, and the facilitatory effects of various musical and non-musical MIT components. However, we suggest that regarding these as competing mechanisms may not be the most fruitful approach to understanding this multi-faceted therapy. Although prior research has aimed to clarify which MIT component and/or mechanism is responsible for its effects, this review advocates for multiple and perhaps synergistically acting mechanisms. Multivariate research methods that can take multiple mechanisms of action into account may be the catalyst for resolving both the ambiguity and some of the existing discrepancies that surround this therapy. A better understanding of not only the individual actions of each component but also the interaction of their related mechanisms would allow further refinements to the MIT protocol to maximize the effectiveness of singing therapy for aphasia.

## Conflict of Interest Statement

The authors declare that the research was conducted in the absence of any commercial or financial relationships that could be construed as a potential conflict of interest.
